# Case Report: Analysis of short-term clinical efficacy of trilaciclib in patients with advanced lung neuroendocrine carcinoma undergoing chemotherapy

**DOI:** 10.3389/fonc.2025.1685910

**Published:** 2025-10-22

**Authors:** Yan Zeng, Nan Lin

**Affiliations:** Department of Oncology, Guiyang Public Health Clinical Center, Guiyang, Guizhou, China

**Keywords:** trilaciclib, chemotherapy, myelosuppression, lung neuroendocrine carcinoma, clinical efficacy

## Abstract

Lung neuroendocrine carcinoma is a rare heterogeneous tumor with the characteristics of high invasiveness, low incidence, and poor survival prognosis. Currently, there is a lack of effective treatment measures, and treatment strategies are mostly extrapolated from small cell lung cancer and non-small cell lung cancer protocols. Studies have shown that more than 55% of patients with extensive-stage small cell lung cancer experience grade 3 or higher myelosuppression after receiving platinum/etoposide-containing chemotherapy, including neutropenia, anemia, and thrombocytopenia. myelosuppression can also increase the risks of infection, bleeding, etc. Severe myelosuppression may delay treatment, reduce dosage, stop medication, and cause other risks that affect tumor prognosis. The results of the study showed that the incidence of grade 3 or higher myelosuppression in patients using trilaciclib (a cyclin-dependent kinase 4/6 [CDK4/6] inhibitor) when receiving platinum-based chemotherapy was significantly lower than that in patients who did not use this drug. In addition, the use of trilaciclib did not affect the anti-tumor effects of chemotherapy and immunotherapy agents during the chemotherapy cycle. This study aimed to explore the effect of trilaciclib on chemotherapy-induced myelosuppression (CIM) in patients with pulmonary neuroendocrine carcinoma undergoing chemotherapy, and to provide a reference for improving patients’ treatment tolerance and quality of life in clinical practice.

## Introduction

Lung neuroendocrine carcinoma (LNEC), primarily comprising small cell lung carcinoma (SCLC) and large cell neuroendocrine carcinoma (LCNEC), carries a poor prognosis. The 5-year overall survival (OS) rates are <15% and 15-25%, respectively. For patients with extensive-stage SCLC (ES-SCLC) and advanced metastatic LCNEC, the median OS is only approximately 1 year, with a 5-year OS rate of 0-5% (1, 2). Current guidelines recommend treating LCNEC with chemotherapy regimens similar to those for SCLC or non-small cell lung cancer (NSCLC). Chemotherapy remains the cornerstone of treatment for advanced lung cancer. Cytotoxic agents damage hematopoietic stem cells (HSCs) in the bone marrow, leading to chemotherapy-induced myelosuppression (CIM). These agents target not only tumor cells but also normal HSCs and progenitor cells, typically manifesting as neutropenia, anemia, and thrombocytopenia (3–5). A recent study reported that over 55% of ES-SCLC patients receiving chemotherapy experienced Grade ≥3 myelosuppressive hematological adverse events (HAEs), with approximately one-third developing Grade ≥3 myelosuppression in two or more lineages (6). Clinically, myelosuppression is associated with an increased risk of infection, hemorrhage, and mortality (7). Severe myelosuppression often necessitates chemotherapy dose reduction, treatment delays, or discontinuation, potentially compromising efficacy and survival outcomes in cancer patients (8).

On February 12, 2021, the FDA approved trilaciclib to reduce the incidence of chemotherapy-induced myelosuppression in adult patients with ES-SCLC receiving platinum/etoposide-containing or topotecan-containing regimens. Trilaciclib is a cyclin-dependent kinase 4/6 (CDK4/6) inhibitor. Its primary mechanism of action involves inducing a transient G1 cell cycle arrest in HSCs. When administered prior to chemotherapy initiation, it protects HSCs from proliferation during cytotoxic chemotherapy exposure, thereby shielding multiple hematopoietic lineages from cytotoxic damage and preserving bone marrow function; The specific mechanism of trilaciclib-induced bone marrow protection is as follows: CDK4/6 inhibitors induce hematopoietic stem cells (HSCs) and hematopoietic progenitor cells (HPCs) in the bone marrow to enter a transient G1-phase cell cycle arrest by inhibiting the activity of cyclin-dependent kinases 4/6 (CDK4/6), temporarily withdrawing them from the actively proliferative phase. When chemotherapeutic agents (which primarily exert cytotoxic effects on cells in the proliferative phase) are administered, hematopoietic cells in the arrested state can evade attack by chemotherapeutic agents, thereby reducing apoptosis and damage of hematopoietic cells. Following the completion of chemotherapy, hematopoietic cells can resume the normal cell cycle progression, preserve the normal hematopoietic function of the bone marrow, and ultimately achieve bone marrow protection (9–11). Studies have demonstrated that trilaciclib reduces the incidence of multilineage myelosuppression in patients receiving etoposide plus platinum-based chemotherapy, exhibits significant myeloprotective effects, and improves healthcare resource utilization (12). Currently, reports on the use of trilaciclib in the antitumor treatment of advanced neuroendocrine carcinoma are scarce. This paper reports a case of first-line treatment for advanced neuroendocrine carcinoma using trilaciclib combined with immunotherapy, anti-angiogenic therapy, and chemotherapy, detailed as follows.

## Case presentation

A 67-year-old male patient, Mr. Geng, presented in January 2024 with cough following exposure to cold, characterized as paroxysmal or intermittent, with expectoration of scant white, thin sputum. Chest CT at a local hospital revealed “multiple bilateral pulmonary lesions.” Symptoms showed no significant improvement after oral cephalosporin antibiotics, and exertional dyspnea developed. Due to delayed medical attention, the patient subsequently developed bilateral lower limb weakness, paralysis requiring bed rest, and impaired consciousness. He was admitted to our hospital on March 11, 2024. Past medical history was unremarkable for hypertension, diabetes mellitus, or coronary heart disease. Family history was non-contributory. He had a 50-pack-year smoking history (average 1 pack/day) and occasional alcohol consumption. ECOG performance status (PS) was 4.

**Figure 1:**
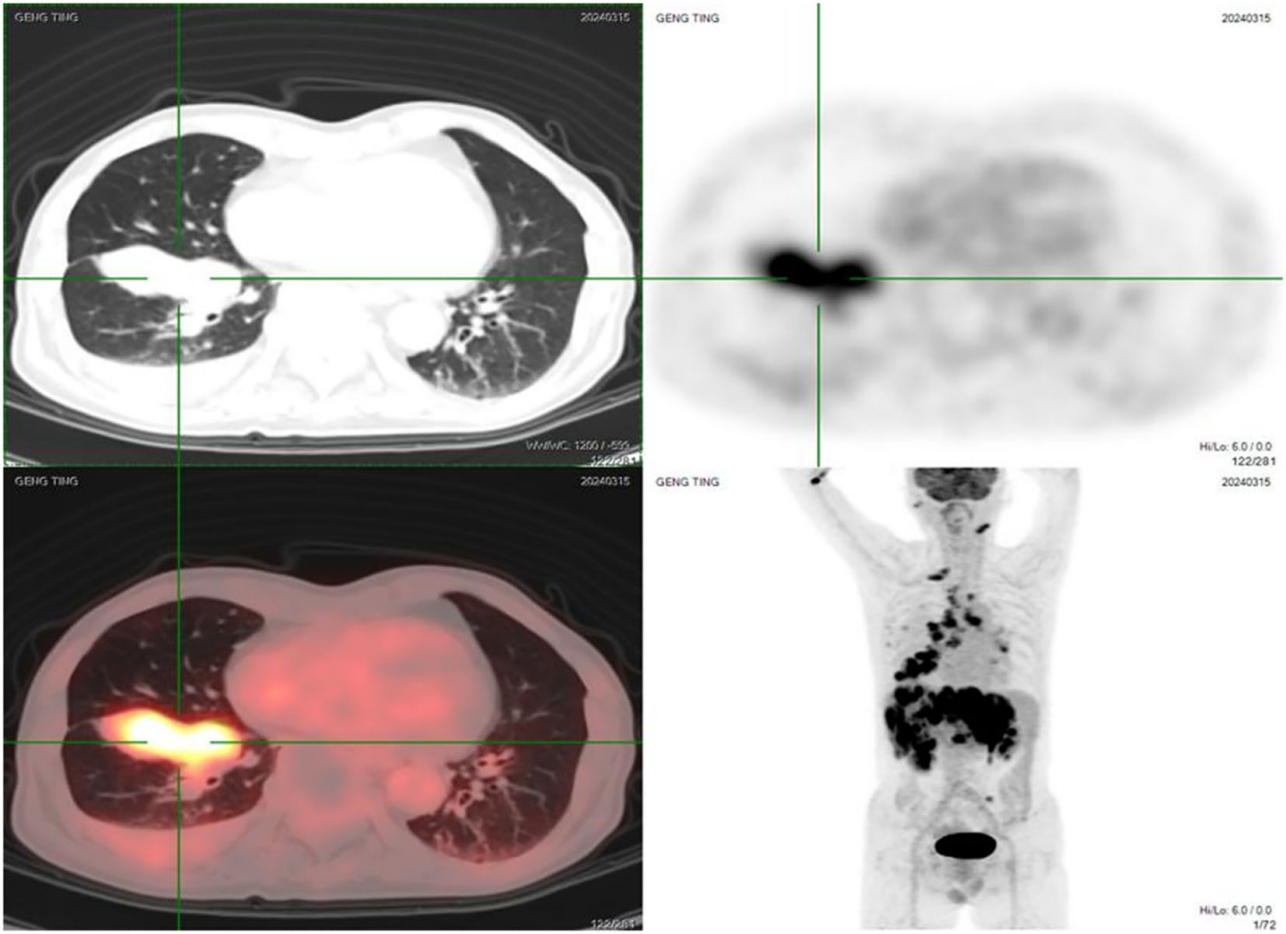
Pre-treatment PET/CT images (March 15, 2024) showing the primary lesion in the right lower lobe, multiple bilateral pulmonary metastases, extensive lymph node metastases, brain metastases, liver metastases, and bone metastases.

On March 13, 2024, a biopsy of a lesion in the right lower lobe was performed. Pathology suggested pulmonary neuroendocrine carcinoma. Positron Emission Tomography/Computed Tomography (PET/CT) on March 15, 2024, revealed:

1. A soft tissue density mass near the hilum in the anteromedial basal segment of the right lower lobe (with corresponding segmental bronchial occlusion), demonstrating increased fluorodeoxyglucose (FDG) uptake, suggestive of central lung cancer.
2. Multiple bilateral pulmonary metastases, with right pulmonary lymphangitis carcinomatosa.
3. Metastatic lymphadenopathy in the right pre-parotid region, left submandibular region (level IB), right supraclavicular fossa (level IV), bilateral hilar regions (10R/L), mediastinum (2R/L, 4R/L, 7), and left mesenteric region.
4. Multiple intracranial metastases.
5. Multiple hepatic metastases.
6. Multiple osseous metastases involving the right sternal end of the clavicle, L2 vertebral body, left sacral ala, left ilium, and right pubis (**Figure 1**).

**Figure 2:**
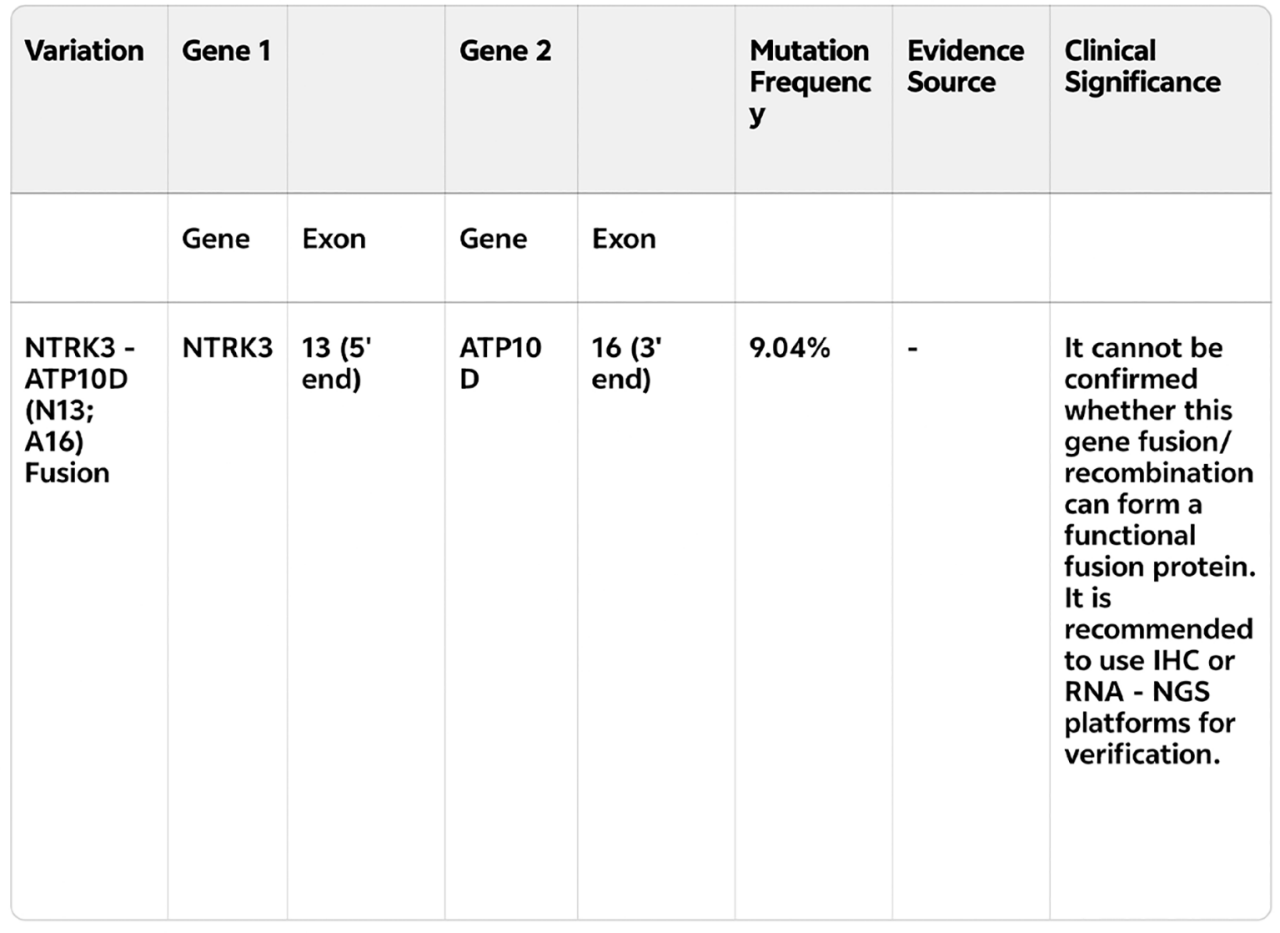
Genetic testing report (English translation of key findings) identifying the NTRK3-ATP10D fusion (mutation frequency: 9.04%).

Lung cancer gene testing on March 24, 2024, identified an NTRK3-ATP10D fusion (mutation frequency: 9.04%) (**Figure 2**).

The clinical diagnosis was: Right lower lung neuroendocrine carcinoma, cT4N3M1c (metastases to multiple lymph nodes, brain, liver, multiple bones) stage IVB (AJCC 8th edition), with NTRK3-ATP10D fusion.

No highly effective standard treatment exists for pulmonary neuroendocrine carcinoma. Current guidelines recommend treating LCNEC with SCLC or NSCLC chemotherapy regimens. Given the patient’s poor general condition and potential intolerance to cisplatin, the ES-SCLC first-line regimen was selected: Etoposide + Carboplatin (EC). After one cycle, the patient responded significantly. From cycle 2 onwards, to enhance efficacy, the regimen was modified to: Etoposide + Carboplatin + Durvalumab + Anlotinib Hydrochloride. The specific treatment plan and timeline are summarized in **Table 1**.

**Table 1:** Treatment timeline and regimen.

Treatment time	Treatment cycle	Treatment regimen and dosage
March 18,2024	Cycle 1	EC chemotherapy:Etoposide 0.1g d1-3, Carboplatin 350mg d1;Trilaciclib 240mg/m²d1-3 (intravenous infusion over 30 minutes within 4 hours before chemotherapy)
April 8,2024	Cycle 2	EC+Durvalumab+Anlotinib Hydrochloride: Etoposide 0.12g d1-3,Carboplatin 350mg d1 (target AUC 3),Durvalumab 1500mg d1,Anlotinib Hydrochloride 8mg d1-14;Denosumab(regular anti-bone metastasis treatment)
April 29,2024	Cycle 3	Same as the treatment regimen and dosage of Cycle 2
May 20,2024	Cycle 4	Same as the treatment regimen and dosage of Cycle 2
June 14,2024	Cycle 5	Same as the treatment regimen and dosage of Cycle 2
July 6,2024	Cycle 6	Same as the treatment regimen and dosage of Cycle 2
June 18,2024- August 1,2024	/	Radiotherapy for intracranial metastases
August 1,2024- October 16,2024	/	Maintenance treatment with Durvalumab + Anlotinib
October 21,2024	/	Chest CT examination showed:The lesion in the right lower lobe enlarged,new metastases appeared in both lungs,some liver metastases enlarged,and intracranial metastases were stable

Cycle 1 (2024-03-18): EC chemotherapy (Etoposide 0.1g d1-3, Carboplatin 350mg d1). Trilaciclib (240mg/m² d1-3, administered within 4 hours before chemotherapy as a 30-minute infusion) was given for myeloprotection.

Cycles 2-6 (2024-04-08, 2024-04-29, 2024-05-20, 2024-06-14, 2024-07-06): EC + Durvalumab + Anlotinib Hydrochloride (Etoposide 0.12g d1-3, Carboplatin 350mg d1 (target AUC 3), Durvalumab 1500mg d1, Anlotinib Hydrochloride 8mg d1-14).

Denosumab was administered regularly for anti-bone metastasis therapy.

**Figure 3:**
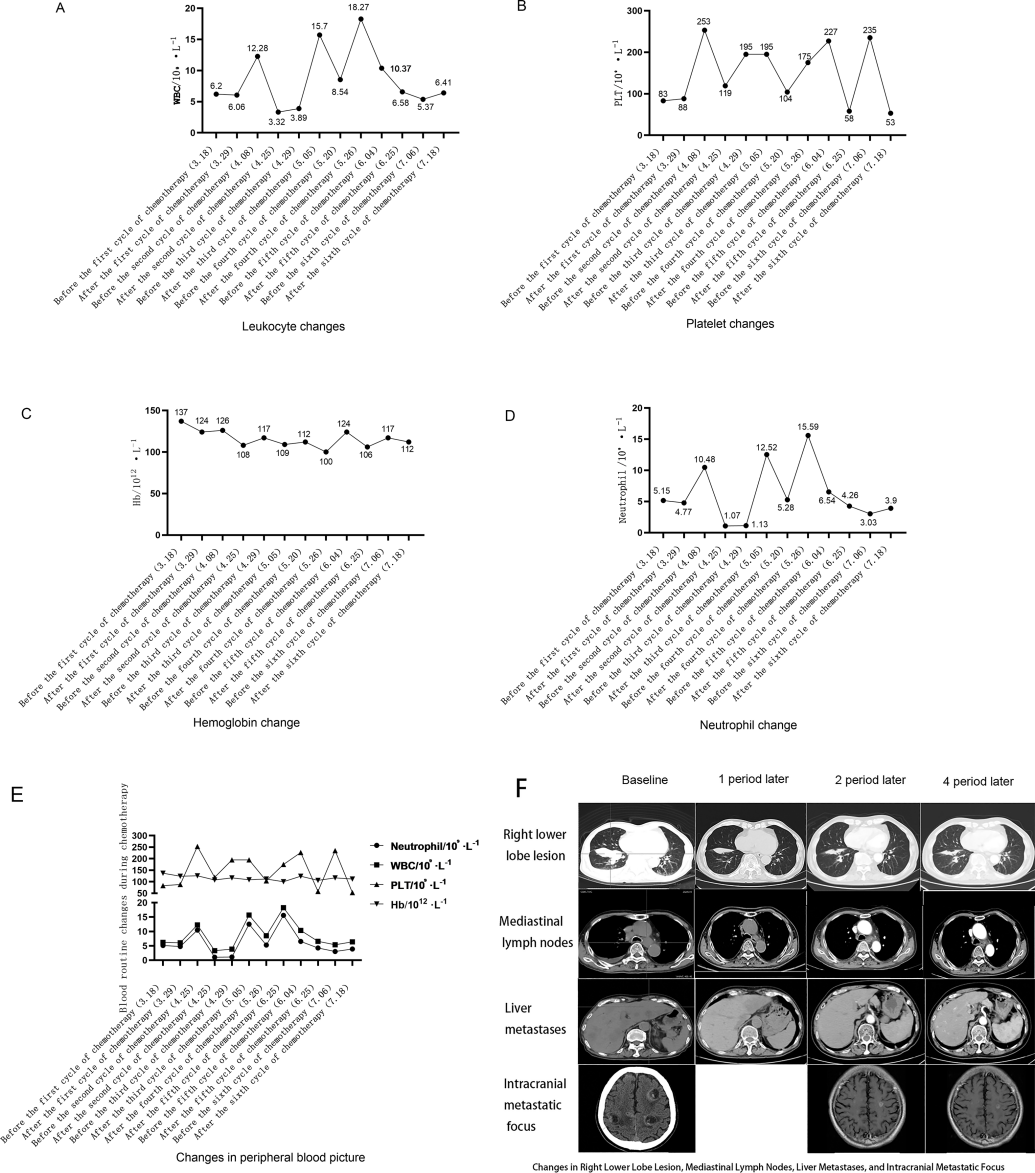
**(A)** Leukocyte count changes during treatment cycles: Leukocyte count trends (unit: ×10^9^/L) at different time points throughout the treatment, including before the first cycle of chemotherapy (March 18, 2024), after the first cycle (March 29), before/after the second to sixth cycles (April 8 to July 18, 2024). No severe leukocyte reduction was observed during the treatment period. **(B)** Platelet count changes during treatment cycles:Platelet count trends (unit: ×10^9^/L) at different time points throughout the treatment, including before the first cycle of chemotherapy (March 18, 2024), after the first cycle (March 29), before/after the second to sixth cycles (April 8 to July 18, 2024). No severe platelet reduction was observed during the treatment period. **(C)** Hemoglobin Level Changes During Treatment Cycles:Hemoglobin level trends (unit: g/L) at different time points throughout the treatment, including before the first cycle of chemotherapy (March 18, 2024), after the first cycle (March 29), before/after the second to sixth cycles (April 8 to July 18, 2024). Only Grade 1 myelosuppression-related hemoglobin fluctuation was observed, with no Grade ≥3 events. **(D)** Neutrophil Count Changes During Treatment Cycles:Neutrophil count trends (unit: ×10^9^/L) at different time points throughout the treatment, showing values such as 1.07, 3.03, and 4.26 at key time nodes (March 18 to July 18, 2024). The neutrophil count remained within a relatively stable range,with no severe neutropenia (Grade ≥3) observed. **(E)** Total Hematological Parameters Changes During Treatment Cycles:Comprehensive trends of key hematological parameters (white blood cells, platelets, hemoglobin, neutrophils; units: ×10^9^/L for white blood cells/platelets/neutrophils, g/L for hemoglobin) at different time points throughout the treatment, All parameters were well-controlled, with only Grade 1 myelosuppression overall. **(F)** Changes in Right Lower Lobe Lesion, Mediastinal Lymph Nodes, Liver Metastases, and Intracranial Metastatic Focus During Treatment:Comparison of the size/status of the right lower lobe lesion, mediastinal lymph nodes, liver metastases, and Intracranial Metastatic Focus. The right lower lobe lesion and mediastinal lymph nodes showed progressive reduction, liver metastases were reduced, and intracranial metastatic foci remained stable, consistent with the partial response (PR) efficacy evaluation.

Under the myeloprotective prophylaxis with trilaciclib, the patient did not develop severe reductions in neutrophils, hemoglobin, or platelets throughout the treatment. Adverse events included Grade 1 myelosuppression (**Figure 3**).

Regarding antitumor efficacy assessment, compared to the baseline PET/CT on March 15, 2024 (pre-immunotherapy/chemotherapy), post-cycle 1 (2024-04-03), post-cycle 2 (2024-04-26), and post-cycle 4 (2024-06-11) imaging showed progressive improvement. Efficacy evaluations at cycles 2 and 4 indicated partial response (PR) (**Figure 3**). Post-cycle 6, all measurable tumor lesions showed minimal change compared to post-cycle 4. The patient’s ECOG PS improved from 4 at baseline to 2 after cycle 1 and 0 after cycle 2. Notably, the patient presented with bilateral lower limb muscle strength of grade 0, paralysis, impaired consciousness, and inability for self-care before treatment. After the first cycle of chemotherapy, bilateral lower limb muscle strength gradually recovered, enabling slow walking and normal responsiveness. Consequently, only chest and upper abdomen CT scans were repeated after cycle 1; brain MRI was not repeated.

## Discussion

Chemotherapy-induced myelosuppression (CIM) is a common and significant adverse reaction in antitumor therapy, leading to complications such as neutropenia, anemia, and thrombocytopenia (3–5). Patients with myelosuppression are more susceptible to infections, sepsis, hemorrhage, reduced quality of life, frequent hospitalizations, and even death (13, 14). Published studies indicate that in Asian populations (China, Korea, Japan), myelosuppression (Grade 3/4 neutropenia: 15.6-93.8%, anemia: 0-33.3%, thrombocytopenia: 0-33.3%) frequently leads to dose reductions or delays in patients with SCLC treated with carboplatin/cisplatin plus etoposide. Limitations on chemotherapy dose intensity may compromise antitumor efficacy; therefore, myelosuppression remains a major side effect requiring immediate attention in lung cancer patients (15–17). Current treatments (e.g., granulocyte colony-stimulating factor [G-CSF] for neutropenia, erythropoiesis-stimulating agents [ESAs] or transfusions for anemia, thrombopoietin receptor agonists [TPO-RAs] or transfusions for thrombocytopenia) only manage symptoms after CIM occurs and do not prevent it. These interventions themselves carry risks: G-CSF can cause bone pain; red blood cell transfusions increase infection risk; ESAs elevate cardiovascular event risk, adding to treatment burden (18, 19). Hence, there is a pressing need for therapies that proactively reduce the incidence of CIM.As the first CDK4/6 inhibitor approved for CIM prevention, Trilaciclib exerts its myeloprotective effect through precise cell cycle regulation: it competitively binds to CDK4/6, inhibits the formation of active complexes between CDK4/6 and cyclin D, and thereby blocks the phosphorylation of retinoblastoma protein (Rb), ultimately arresting hematopoietic stem cells (HSCs) and hematopoietic progenitor cells (HPCs) in the G1 phase (11, 12). This phase has extremely low sensitivity to chemotherapeutic agents (such as platinum and etoposide) that target proliferating cells, and the arrest is reversible—after chemotherapy, CDK4/6 activity can quickly recover, allowing HSCs and HPCs to restart the normal cell cycle and hematopoietic function, reducing damage to hematopoietic cells at the source. This is fundamentally different from “*post-hoc* remedial” drugs like granulocyte colony-stimulating factor (G-CSF) (18). In addition, preliminary studies have suggested that Trilaciclib’s inhibitory effect on CDK4/6 may alleviate chemotherapy-induced damage to T cells and natural killer (NK) cells, optimizing the immune microenvironment for “chemotherapy + immunotherapy” combination regimens. However, the specific effect of this role in neuroendocrine carcinoma still requires further verification. For lung neuroendocrine carcinoma (LNEC), the application value of Trilaciclib is highly consistent with the disease’s characteristics. Small cell lung cancer (SCLC) and large cell neuroendocrine carcinoma (LCNEC)—the main subtypes of LNEC—both rely on chemotherapy as the core treatment. Studies have shown that more than 55% of patients with extensive-stage SCLC (ES-SCLC) develop grade ≥3 CIM after chemotherapy (6). Trilaciclib can reduce the risk of chemotherapy dose reduction and treatment delay, ensuring adequate chemotherapy to maintain efficacy (12). At the same time, most LNEC patients are elderly (such as the 67-year-old patient in this case) and often have smoking-related underlying diseases, resulting in poor tolerance to CIM. Trilaciclib can significantly reduce the incidence of grade ≥3 neutropenia and thrombocytopenia (20, 21). Just as the patient in this case only developed grade 1 myelosuppression, it effectively avoids the vicious cycle of “complications - treatment interruption - tumor progression” (7, 8). Furthermore, although the “chemotherapy + immunotherapy + anti-angiogenic therapy” combination regimen currently used in LNEC treatment can improve efficacy, it has prominent toxicity overlap issues (e.g., the incidence of grade ≥3 adverse reactions in the four-drug combination regimen reaches 93.1%) (22). Trilaciclib can reduce the toxicity burden without affecting the anti-tumor effect of the combination regimen (the patient in this case achieved partial remission with a progression-free survival of 7 months) (22). Moreover, compared with G-CSF, which only targets a single lineage, Trilaciclib can protect neutrophils, erythrocytes, and platelets simultaneously, filling the gap in multi-lineage myeloprotection for LNEC (12). It particularly provides chemotherapy safety support for subtypes with limited access to targeted drugs, such as NTRK fusion-positive LNEC.

The pathological subtype in this case was neuroendocrine carcinoma with left submandibular lymph node metastasis. Further distinction between SCLC and LCNEC was not possible; thus, an SCLC treatment approach was adopted. Based on the ETER701 study, which demonstrated improved outcomes in ES-SCLC with chemoimmunotherapy combined with the anti-angiogenic agent anlotinib (median OS extended to 19.3 months *vs*. 11.9 months with EC alone; median PFS 6.9 months *vs*. 4.2 months), a four-drug combination regimen was selected for this patient.

Trilaciclib, the first FDA-approved myeloprotective agent, received approval in China in July 2022 for administration prior to platinum/etoposide-containing or topotecan-containing regimens in ES-SCLC. As a competitive CDK4/6 inhibitor, it reversibly induces G1 cell cycle arrest, protecting hematopoietic lineages from chemotherapy-induced DNA damage and reducing the incidence of single-lineage and multilineage Grade ≥3 CIM in ES-SCLC patients (9–11). Studies indicate that CIM-related toxicities are more common in elderly patients and may be associated with poorer treatment outcomes (20, 21). Trilaciclib proactively protects HSCs, thereby benefiting all hematopoietic lineages. In this case, the patient completed all six planned chemotherapy cycles while receiving trilaciclib. Antitumor efficacy was assessed as PR, with no chemotherapy dose reductions or treatment delays. Quality of life and mental status also improved. This demonstrates that trilaciclib, used prior to chemoimmunotherapy combined with anti-angiogenic therapy, provides effective and safe myeloprotection in elderly patients receiving first-line treatment for advanced LNEC. By enhancing tolerance to chemotherapy, trilaciclib enabled the patient to complete the full course of antitumor treatment on schedule and at planned doses, achieving stable disease control.

The ETER701 study also reported increased adverse event rates with the four-drug combination, with hematological toxicity (thrombocytopenia, neutropenia, leukopenia) being the most common. Grade ≥3 adverse events occurred in 93.1% of the four-drug group *vs*. 87.0% in the EC-alone group. Notably, this patient had a baseline ECOG PS of 4, which improved to 2 after the first EC cycle before initiating the four-drug combination. While receiving trilaciclib, the highest hematological toxicity observed was Grade 2, with no Grade ≥3 hematological adverse events.

Subsequent treatment included radiotherapy for intracranial metastases from June 18, 2024, to August 1, 2024, followed by maintenance therapy with Durvalumab + Anlotinib until October 16, 2024. A chest CT on October 21, 2024, showed enlargement of the right lower lobe lesion, new bilateral pulmonary metastases, enlargement of some hepatic metastases, and stable intracranial metastases. Progression-free survival (PFS) was 7 months, consistent with the ETER701 study. In summary, this case report preliminarily confirms the myeloprotective benefit of administering trilaciclib prior to chemotherapy without compromising antitumor efficacy.

Currently, few therapies proactively prevent CIM before chemotherapy initiation. Clinical interventions like ESAs, G-CSF, recombinant human thrombopoietin (rhTPO), or blood component transfusions are typically introduced after chemotherapy-induced hematopoietic damage and do not prevent CIM. In clinical practice, primary prophylaxis with G-CSF is recommended when the risk of febrile neutropenia (FN) is assessed as high (>20%), proven to reduce FN risk and early mortality without significantly impacting antitumor response (23, 24). However, these reactive measures are not always fully effective and may themselves lead to suboptimal protection, treatment delays, prolonged hospitalization, treatment discontinuation, reduced quality of life, and increased financial burden (22, 25). This patient did not require blood component transfusions, G-CSF, or rhTPO during trilaciclib-protected cycles, except for rhTPO administration for Grade II thrombocytopenia occurring only in cycles 2 and 5. This indicates trilaciclib enhanced multilineage myeloprotection and reduced the incidence of Grade ≥3 hematological toxicity.

Research suggests trilaciclib may positively impact outcomes by preventing CIM, preserving immune system function, and minimizing cytotoxic adverse events, although many findings remain to be fully elucidated. While this drug does not appear to compromise the efficacy of chemoimmunotherapy combined with anti-angiogenic agents, whether it contributes to prolonged survival or improved quality of life is still debated and requires further investigation.

This study is a single-case report, which inherently limits the generalizability of the findings. The outcomes observed may be influenced by unique patient characteristics and could be subject to chance. While the results are promising, they necessitate validation through larger, prospective clinical trials in patients with advanced lung neuroendocrine carcinoma to confirm the myeloprotective efficacy and safety profile of trilaciclib in this specific population.

In conclusion, the findings from this case report provide initial evidence suggesting the myeloprotective benefit of administering trilaciclib prior to chemotherapy in pulmonary neuroendocrine carcinoma, manifested as a clinically meaningful reduction in CIM, without adversely affecting antitumor efficacy.

## Data Availability

The original contributions presented in the study are included in the article/supplementary material. Further inquiries can be directed to the corresponding author.
